# *In vitro* activity of isavuconazole, ravuconazole, and comparison of the Sensititre YeastOne and CLSI broth microdilution methods against clinical isolates of *Trichosporon* species

**DOI:** 10.1128/jcm.00319-25

**Published:** 2025-05-12

**Authors:** Shih-Hao Lo, Yi-Ting Tseng, Yee-Chun Chen, Mao-Wang Ho, Chen-Hsiang Lee, Po-Liang Lu, Shang-Yi Lin

**Affiliations:** 1 Department of Internal Medicine, Kaohsiung Municipal Siaogang Hospital, Kaohsiung Medical University 38023, Kaohsiung, Taiwan; 2 Division of Infectious Diseases, Department of Internal Medicine, Kaohsiung Medical University Hospital, Kaohsiung Medical University 38023, Kaohsiung, Taiwan; 3 Department of Internal Medicine, National Taiwan University Hospital and College of Medicine 38006, Taipei, Taiwan; 4 Division of Infectious Diseases, Department of Internal Medicine, China Medical University Hospital 38020, Taichung, Taiwan; 5 Department of Internal Medicine, Kaohsiung Chang Gung Memorial Hospital, College of Medicine, Chang Gung University 71589, Kaohsiung, Taiwan; 6 Department of post-baccalaureate Medicine, Kaohsiung Medical University 38023, Kaohsiung, Taiwan; 7 Center for Liquid Biopsy and Cohort Research, Kaohsiung Medical University 38023, Kaohsiung, Taiwan; 8 Department of Laboratory Medicine, Kaohsiung Medical University Hospital, Kaohsiung Medical University 38023, Kaohsiung, Taiwan; 9 Graduate Institute of Clinical Medicine, College of Medicine, Kaohsiung Medical University 38023, Kaohsiung, Taiwan; University of Utah, Salt Lake City, USA

**Keywords:** *Trichosporon*, isavuconazole, ravuconazole, voriconazole, fluconazole, Sensititre YeastOne, CLSI, minimal inhibitory concentration

## Abstract

This study aimed to evaluate the *in vitro* activity of isavuconazole and ravuconazole against clinical *Trichosporon* isolates. Additionally, we assessed the performance of the Sensititre YeastOne (SYO) assay compared to the reference Clinical and Laboratory Standards Institute (CLSI) broth microdilution (BMD) method for antifungal susceptibility testing. A total of 267 *Trichosporon* clinical isolates were collected from multiple centers in Taiwan between 2008 and 2020. The MIC values for amphotericin B, fluconazole, itraconazole, posaconazole, and voriconazole obtained using the CLSI BMD were compared to those determined using SYO. Among the seven antifungal agents tested using the CLSI BMD method, *T. asahii* isolates (*n* = 224) exhibited significantly higher MICs for fluconazole, voriconazole, isavuconazole, and ravuconazole compared to non-*T*. *asahii* isolates (*n* = 43) (*p* < 0.05). Across all isolates, voriconazole demonstrated the lowest geometric mean (GM) MIC (0.06 mg/L). For *T. asahii*, the MIC₅₀, MIC₉₀, and GM MICs for posaconazole, isavuconazole, and ravuconazole were within ±1 twofold dilution, while mode MICs showed slightly greater variation (MIC₅₀, MIC₉₀, mode, and GM MICs were 0.25, 0.5, 0.5, and 0.28 mg/L; 0.12, 0.5, 0.12, and 0.17 mg/L; and 0.25, 0.5, 0.25, and 0.21 mg/L, respectively). The consistency of the MIC measurements between the SYO and CLSI BMD methods was assessed for amphotericin B, fluconazole, itraconazole, posaconazole, and voriconazole. The highest agreement within ±1 twofold dilution for *T. asahii* isolates was observed for posaconazole (94.2%). The essential agreement within ±2 twofold dilutions between both methods for all drugs was >97%. In conclusion, isavuconazole and ravuconazole demonstrated favorable *in vitro* activity against clinical *Trichosporon* isolates, while the SYO assay showed good concordance with the CLSI BMD method.

**IMPORTANCE:**

Invasive infections caused by *Trichosporon* species pose significant therapeutic challenges, primarily due to their intrinsic resistance to echinocandins and the limited availability of effective treatment options. This study provides essential data on the *in vitro* activity of newer azoles and comprehensively evaluates the performance and concordance of the Sensititre YeastOne (SYO) and CLSI broth microdilution (BMD) methods. The current study analyzed 267 *Trichosporon* clinical isolates collected from multicenter settings in Taiwan. Our results demonstrate that both isavuconazole and ravuconazole exhibit favorable *in vitro* activities against *Trichosporon* species. For *T. asahii*, the essential agreement between the SYO and CLSI BMD methods exceeded 97% for all tested antifungal agents, indicating that the SYO method exhibits good concordance for most *Trichosporon* species. Further investigations are warranted to validate these findings and to assess their clinical implications.

## INTRODUCTION

*Trichosporon*, which belongs to the same phylum as *Basidiomycota* as *Cryptococcus*, is a yeast-like fungal pathogen commonly found in soil, decaying matter, plants, and water. Moreover, it is part of the resident and transient microbiota of the human oral, cutaneous, and intestinal flora (1–4). *Trichosporon* species have been recognized as opportunistic pathogens capable of causing life-threatening invasive diseases, particularly in immunocompromised individuals (5, 6). A multicenter surveillance study in Asia identified *Trichosporon* spp. as the second most common pathogen in patients with non-*Candida* yeast fungemia (7). Moreover, a recent study reported a high mortality rate (80%) in patients with severe COVID-19 and *Trichosporon* fungemia coinfections (8).

Treatment of invasive *Trichosporon* disease remains a challenge due to the lack of randomized clinical trials and large-scale cohort studies. Currently, voriconazole and posaconazole are the preferred therapeutic options for *Trichosporon* infections due to their relatively low minimal inhibitory concentrations (MICs) and supporting clinical data from retrospective studies (5, 9). Isavuconazole, a second-generation broad-spectrum triazole, is active against yeasts, dimorphic fungi, and molds while offering a favorable safety profile and predictable pharmacokinetics (10), with a few case series reporting its potential clinical efficiency (11–13). However, studies evaluating the *in vitro* activity of isavuconazole against *Trichosporon* species are limited (14, 15). Similarly, ravuconazole, an extended-spectrum triazole agent with demonstrated *in vitro* efficacy against *Candida*, *Aspergillus*, *Cryptococcus*, and dematiaceous fungi, was approved for use in Japan as an oral treatment for onychomycosis in 2018 (16). However, data regarding its *in vitro* activity against *Trichosporon* spp. are limited.

Several techniques are available for fungi MIC testing, including broth microdilution (BMD) reference methods recommended by the Clinical and Laboratory Standards Institute (CLSI) and the European Committee on Antimicrobial Susceptibility Testing (EUCAST) (17, 18). Despite their reliability, BMD methods are labor-intensive, time-consuming, and typically limited to central laboratories. Commercial methods, such as the Sensititre Yeast One (SYO) assay and agar-based methods (e.g., Etest), offer standardized and efficient alternative MIC testing methods suitable for routine clinical microbiology laboratories. The SYO assay uses a colorimetric microdilution method and has been approved by the US Food and Drug Administration for use with *Candida* species (19, 20). Only a few studies have reported the MICs for *Trichosporon* species based on SYO assays (6, 21, 22). However, the agreement between the CLSI reference BMD method and the SYO assay remains unclear.

In the current study, we evaluated the *in vitro* activity of isavuconazole and ravuconazole against a large collection of clinical *Trichosporon* isolates from multiple centers in Taiwan using the CLSI BMD method. Additionally, we assessed the performance and concordance of the SYO and BMD methods to determine the *in vitro* antifungal activities of amphotericin B, fluconazole, itraconazole, voriconazole, and posaconazole against *Trichosporon* species.

## MATERIALS AND METHODS

### *Trichosporon* isolates

A total of 267 *Trichosporon* clinical isolates were collected from four medical centers in Taiwan, including the National Taiwan University Hospital, the China Medical University Hospital, the Kaohsiung Medical University Hospital, and the Kaohsiung Chang Gung Memorial Hospital, between 2008 and 2020. These isolates were initially identified using standard laboratory procedures, including the presence of phenotypic characteristics, the API 32C (bioMérieux Vitek, Marcy l'Etoile, France), the VITEK 2 system (bioMérieux, Hazelwood, MO, USA), and the MALDI Bio-Typer (Bruker, Daltonik GmbH, Bremen, Germany). Molecular confirmation was subsequently performed at Kaohsiung Medical University Hospital by sequencing the internal transcribed spacer (ITS) and intergenic spacer 1 (IGS1) regions of ribosomal DNA (6, 23). This study was reviewed and approved by the Institutional Review Board (IRB No. KMUHIRB-E(II)−20250010).

### Antifungal susceptibility testing

#### CLSI broth microdilution reference method

Antifungal susceptibility testing (AST) for fluconazole, itraconazole, voriconazole, posaconazole, ravuconazole, amphotericin B (Sigma, St. Louis, MO, USA), and isavuconazole (Pfizer) was performed using the BMD method according to the CLSI document M27, 4th edition (17). MIC values were determined after 24–48 hours of incubation, depending on the growth observed in the positive control wells (17, 24). The drug concentration ranges tested were as follows: 0.25–256 mg/L for fluconazole, 0.015–16 mg/L for itraconazole, and 0.008–8 mg/L for voriconazole, posaconazole, ravuconazole, isavuconazole, and amphotericin B. Quality control was performed for each experiment using *Candida krusei* ATCC 6258 and *Candida parapsilosis* ATCC 22019 reference strains.

#### Sensititre YeastOne assay

The SYO Y10 (Trek Diagnostic Systems, Ltd., East Grinstead, UK) was used for AST, and the reading and interpretation were performed according to the manufacturer’s instructions. The MIC was defined as the lowest concentration of the antifungal agents at which the color in the well changed from red to blue (negative, indicating no growth), except for amphotericin B, which was defined as the lowest concentration that had no color change. The tested concentration ranges were as follows: fluconazole (0.12–256 mg/L), itraconazole (0.015–16 mg/L), posaconazole (0.008–8 mg/L), voriconazole (0.008–8 mg/L), and amphotericin B (0.12–8 mg/L) (25, 26).

#### Data analysis

The MICs for fluconazole, itraconazole, posaconazole, voriconazole, and amphotericin B were compared using the BMD and SYO assays. A local epidemiological cutoff value (L-ECOFF) was established to assess the categorical agreement between the reference (BMD) and commercial (SYO) methods. L-ECOFF, which represents the upper MIC limit of the wild-type (WT) distribution among the tested isolates, was determined using the ECOFFinder software (https://clsi.org/meetings/susceptibility-testing-subcommittees/ecoffinder/). For *T. asahii*, the epidemiological cutoff values (ECVs) provided by CLSI M57S were 1 mg/L for amphotericin B, posaconazole, and itraconazole and 8 mg/L for fluconazole (27). Isolates were classified as WT if their MIC was ≤L-ECOFF or CLSI-ECV and non-WT if their MIC exceeded the L-ECOFF or CLSI-ECV. The concordance between the BMD and SYO results was assessed using essential agreement (EA) and categorical agreement (CA). EA was defined as MIC values obtained by both methods agreeing within ±2 log_2_ dilutions. The CA refers to the percentage of isolates classified into the same category (WT or non-WT) using both methods.

All statistical analyses were performed using SPSS version 25 (IBM Corp., Armonk, NY, USA). MIC values were log-transformed, and statistical differences between the methods were evaluated using a paired *t*-test. The median and range of the log₂ MIC differences between the two methods were calculated. A *P*-value < 0.05 was considered statistically significant.

## RESULTS

A total of 267 *Trichosporon* isolates were enrolled in this study. Most isolates were obtained from the bloodstream (39.3%) (Table S1). Among these, 224 isolates (83.9%) were identified as *T. asahii*, followed by 11 *T. mucoides* (4.1%), 11 *T. montevideense* (4.1%), 7 *T. faecale* (2.6%), 6 *T. dermatis* (2.2%), 3 *T. japonicum* (1.1%), 2 *T. mycotoxinivorans* (0.7%), and 1 isolate each of *T. dohaense*, *T. inkin*, and *T. jirovecii* (0.4% each).

Table 1 summarizes the *in vitro* activity of amphotericin B, fluconazole, itraconazole, posaconazole, voriconazole, ravuconazole, and isavuconazole against 267 *Trichosporon* isolates using BMD and SYO assays. *T. asahii* isolates (*n* = 224) exhibited significantly higher MICs for fluconazole, voriconazole, isavuconazole, and ravuconazole than non-*T*. *asahii* isolates (*n* = 43) (*p* < 0.05). Overall, voriconazole showed the most potent activity and exhibited high MICs in both methods, with the respective MIC ranges of MIC_50_, MIC_90_, mode, and geometric mean (GM) MIC. For *T. asahii*, the MIC₅₀, MIC₉₀, and GM MICs for posaconazole, isavuconazole, and ravuconazole were within ±1 twofold dilution, while mode MICs showed slightly greater variation (MIC₅₀, MIC₉₀, mode, and GM MICs were 0.25, 0.5, 0.5, and 0.28 mg/L; 0.12, 0.5, 0.12, and 0.17 mg/L; and 0.25, 0.5, 0.25, and 0.21 mg/L, respectively). For non-*T. asahii*, the mode and GM MICs of posaconazole were higher than those of isavuconazole and ravuconazole. Two *T. mycotoxinivorans* isolates demonstrated high MIC values for azole antifungal agents (Table S2).

**TABLE 1:** *In vitro* activity of antifungal agents against 267 *Trichosporon* isolates^*a*^ using the CLSI broth microdilution and the SYO methods^*b*^^,^^*c*^

Antifungal agent	Methods	MIC range	MIC_50_	MIC_90_	Mode MIC	GM	L-ECOFF
		mg/L					
Amphotericin B							
*T. asahii*	CLSI	0.25–2.0	1	1	1	0.9	4
*T. asahii*	SYO	0.12–2.0	0.5	1	0.5	0.48	2
Non-*T*. *asahii*	CLSI	0.12–2.0	1	1	1	0.78	–
Non-*T*. *asahii*	SYO	0.12–2.0	0.5	1	0.5	0.47	–
Fluconazole							
*T. asahii*	CLSI	1.0–32.0	4	8	4	4.18	16
*T. asahii*	SYO	0.5–32.0	4	8	4	5.81	16
Non-*T*. *asahii*	CLSI	1.0–16.0	2	4	4	2.55	–
Non-*T*. *asahii*	SYO	0.12–64.0	4	8	4	2.8	–
Itraconazole							
*T. asahii*	CLSI	0.25–1.0	0.5	0.5	0.5	0.48	2
*T. asahii*	SYO	0.06–1.0	0.25	0.5	0.25	0.24	1
Non-*T*. *asahii*	CLSI	0.06–1.0	0.5	0.5	0.5	0.36	–
Non-*T*. *asahii*	SYO	0.008–0.5	0.12	0.25	0.12	0.11	–
Posaconazole							
*T. asahii*	CLSI	0.03–1.0	0.25	0.5	0.25	0.28	1
*T. asahii*	SYO	0.12–1.0	0.25	0.5	0.25	0.32	1
Non-*T*. *asahii*	CLSI	0.03–0.5	0.25	0.5	0.25	0.22	–
Non-*T*. *asahii*	SYO	0.008–1.0	0.12	0.5	0.12	0.15	–
Voriconazole							
*T. asahii*	CLSI	0.015–0.5	0.06	0.12	0.06	0.06	0.25
*T. asahii*	SYO	0.03–0.5	0.12	0.25	0.12	0.12	0.5
Non-*T*. *asahii*	CLSI	0.015–0.5	0.03	0.25	0.03	0.05	–
Non-*T*. *asahii*	SYO	0.008–1.0	0.06	0.25	0.12	0.07	–
Ravuconazole							
*T. asahii*	CLSI	0.03–2.0	0.25	0.5	0.25	0.21	1
Non-*T*. *asahii*	CLSI	0.008–1	0.12	0.5	0.03	0.11	_
Isavuconazole							
*T. asahii*	CLSI	0.03–0.5	0.12	0.5	0.12	0.17	0.5
Non-*T*. *asahii*	CLSI	0.015–2.0	0.06	0.25	0.03	0.08	_

^*a*^ Included 224 *T. asahii* isolates and 43 non-*T. asahii isolates*.

^*b*^ MIC, minimal inhibitory concentration; CLSI, Clinical and Laboratory Standards Institute; SYO, Sensititre YeastOne; GM, geometric mean; L-ECOFF, Local epidemiological cut-off value.

^*c*^ –, indicates that the value could not be calculated.

**Fig 1:**
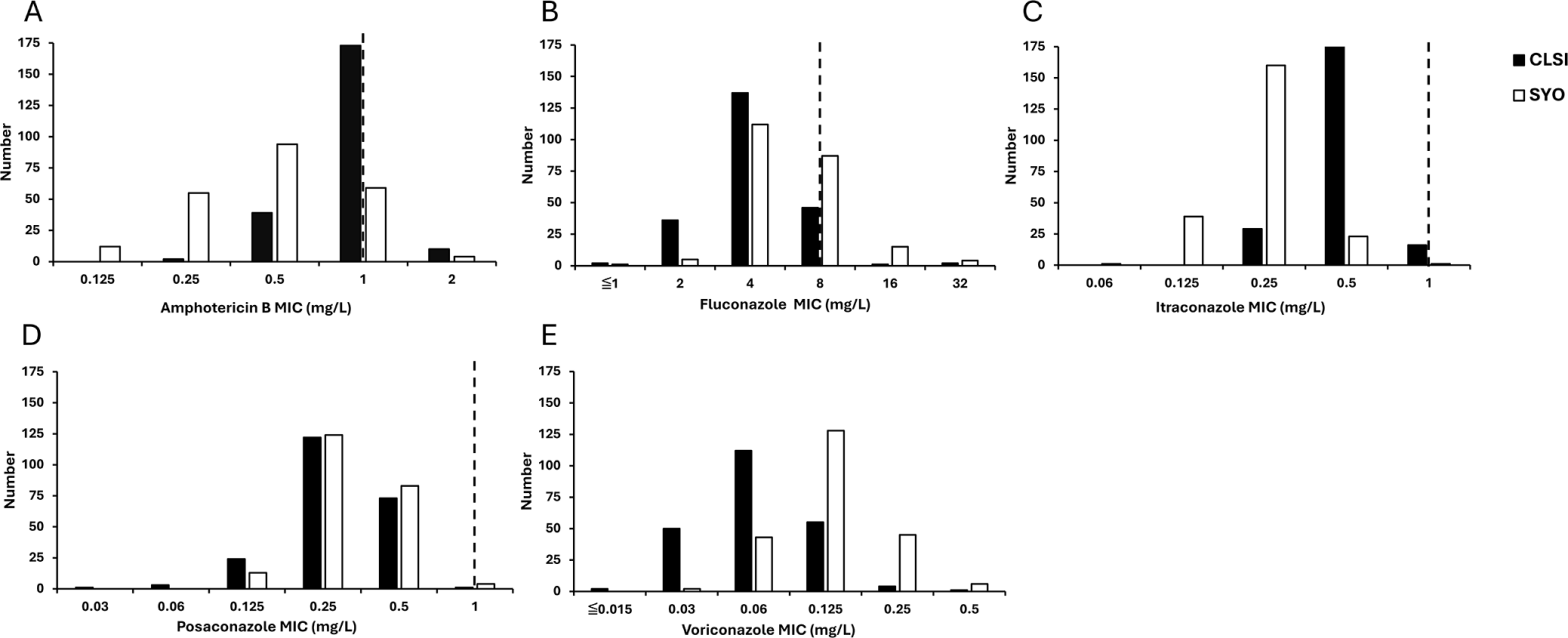
CLSI and Sensititre YeastOne (SYO) MIC distributions of amphotericin B (A), fluconazole (B), itraconazole (C), posaconazole (D), and voriconazole (E) for 224 *T. asahii* isolates. Dashed lines represent ECVs provided by CLSI M57S.

For *T. asahii* isolates (Table 1), the GM MICs for amphotericin B and itraconazole obtained using the SYO method were approximately one dilution lower than those determined by the BMD method. Conversely, the GM MICs for voriconazole were one dilution higher when using the SYO assay than the BMD method. The MIC distributions of *T. asahii* isolates obtained using the SYO and CLSI BMD are presented in Fig. 1. The L-ECOFFs for isavuconazole and ravuconazole against *T. asahii* were 0.5 mg/L and 1 mg/L, respectively (Table 1) (Fig. S1). Overall, the SYO and CLSI BMD methods yielded comparable L-ECOFFs (within one dilution) for all drug methods. (Fig. S1 and S2).

The consistency of the MIC measurements between the SYO and CLSI BMD methods was assessed for amphotericin B, fluconazole, itraconazole, posaconazole, and voriconazole (Table 2). For *T. asahii* isolates, the highest agreement within ±1 twofold dilution was observed for posaconazole (94.2%). The essential agreement within ±2 twofold dilutions between both methods for all drugs was >97%. For non-*T*. *asahii* isolates, the agreement within 2 twofold dilutions between both methods for all drugs was >90%, with the exception of itraconazole (79.1%). Most non-*T*. *asahii* species demonstrated high consistency (≥90%) for both methods (Table S3).

**TABLE 2:** The agreement between the SYO and the CLSI BMD methods^*a*^

		No. (%) of isolates for which MICs determined by SYO differed from MICs determined by the CLSI BMD method at the indicated dilution							Agreement within (%)	
	No.	≥3	2	1	0	−1	−2	≤−3	±1 dilution	±2 dilution
Amphotericin B										
*T. asahii*	224	0	1 (0.4)	7 (3.1)	60 (26.8)	105 (46.9)	46 (20.5)	5 (2.2)	172 (76.8)	219 (97.8)
Non-*T*. *asahii*	43	1 (2.3)	0	2 (4.7)	13 (30.2)	19 (44.2)	6 (14.0)	2 (4.7)	34 (79.1)	40 (93.0)
Fluconazole										
*T. asahii*	224	2 (0.9)	19 (8.5)	81 (36.2)	106 (47.3)	15 (6.7)	0	1 (0.4)	202 (90.2)	221 (98.7)
Non-*T*. *asahii*	43	0	4 (9.3)	11 (25.6)	20 (46.5)	5 (11.6)	2 (4.7)	1 (2.3)	36 (83.7)	42 (97.7)
Itraconazole										
*T. asahii*	224	0	1 (0.4)	0	43 (19.2)	132 (58.9)	47 (21.0)	1 (0.4)	175 (78.1)	223 (99.6)
Non-*T*. *asahii*	43	0	1 (2.3)	2 (4.7)	8 (18.6)	8 (18.6)	15 (34.9)	9 (20.9)	18 (41.9)	34 (79.1)
Posaconazole										
*T. asahii*	224	2 (0.9)	8 (3.6)	63 (28.1)	105 (46.9)	43 (19.2)	3 (1.3)	0	211 (94.2)	222 (99.1)
Non-*T*. *asahii*	43	0	1 (2.3)	9 (20.9)	10 (23.3)	15 (34.9)	5 (11.6)	3 (7.0)	34 (79.1)	40 (93.0)
Voriconazole										
*T. asahii*	224	5 (2.2)	43 (19.2)	124 (55.4)	49 (21.9)	3 (1.3)	0	0	176 (78.6)	219 (97.8)
Non-*T*. *asahii*	43	3 (7.0)	9 (20.9)	9 (20.9)	12 (27.9)	7 (16.3)	3 (7.0)	0	28 (65.1)	40 (93.0)

^*a*^ CLSI, Clinical and Laboratory Standards Institute; BMD, broth microdilution; SYO, Sensititre YeastOne; MIC, minimal inhibitory concentration.

Table 3 shows the EA and CA of the antifungal agents between the two methods for *T. asahii* isolates. The EA and CA of all antifungal agents exceeded 97% when assessed using L-ECOFF. However, when the CLSI-established ECVs were applied, the CA for amphotericin B and fluconazole slightly decreased to 93.8% and 92.9%, respectively.

**TABLE 3:** Agreement between the SYO and CLSI BMD results for *Trichosporon asahii* based on the local ECOFF and ECV provided by CLSI M57S**^*a*,*b*^**

	Method	L-ECOFF obtained by indicated method (%)			
		Wild type	Non-wild type	EA	CA
Amphotericin B	CLSI BMD	224 (100)	0 (0)	97.8	100
	SYO	224 (100)	0 (0)		
Fluconazole	CLSI BMD	222 (91.1)	2 (8.9)	98.7	99.1
	SYO	220 (98.7)	4 (1.3)		
Itraconazole	CLSI BMD	224 (100)	0 (0)	99.6	100
	SYO	224 (100)	0 (0)		
Posaconazole	CLSI BMD	224 (100)	0 (0)	99.1	100
	SYO	224 (100)	0 (0)		
Voriconazole	CLSI BMD SYO	223 (99.6) 224 (100)	1 (0.4) 0 (0)	97.8	97.8

	Method	ECV^*a*^ (%)			
		Wild type	Non-wild type	EA	CA
Amphotericin B	CLSI BMD	214 (95.5)	10 (4.5)	97.8	93.8
	SYO	220 (98.2)	4 (1.8)		
Fluconazole	CLSI BMD	221 (98.7)	3 (1.3)	98.7	92.9
	SYO	205 (91.5)	19 (8.5)		
Itraconazole	CLSI BMD	224 (100)	0 (0)	99.6	100
	SYO	224 (100)	0 (0)		
Posaconazole	CLSI BMD	224 (100)	0 (0)	99.1	100
	SYO	224 (100)	0 (0)		

^*a*^ The cutoff values were provided by CLSI M57S (27).

^*b*^ BMD, broth microdilution; CA, categorical agreement; EA, essential agreement; ECV, epidemiological cutoff value; SYO, Sensititre YeastOne.

## DISCUSSION

In the current study, we assessed the *in vitro* activity of antifungal agents and evaluated the agreement between the SYO and CLSI BMD methods using a large collection of clinical *Trichosporon* isolates obtained from multiple centers in Taiwan. Our data demonstrate that voriconazole exhibits the highest activity among the tested triazoles against *Trichosporon* species. Furthermore, isavuconazole, ravuconazole, and posaconazole displayed similar and favorable *in vitro* activities against *Trichosporon* isolates. For *T. asahii*, the EA within ±2 twofold dilutions between the SYO and CLSI BMD methods was higher than 97% for all antifungal agents, indicating that the SYO method had good concordance for most *Trichosporon* species.

Our findings indicated that the triazoles exhibit better drug susceptibility against *Trichosporon* species, with voriconazole demonstrating the lowest MIC_50_, MIC_90_, mode, and GM MIC values among the tested antifungal agents (6, 15, 24, 28). For *T. asahii*, the MICs of posaconazole, isavuconazole, and ravuconazole were similar within a ±1 twofold dilution (Table 1). These results are consistent with recent studies highlighting the favorable *in vitro* activity of isavuconazole against most clinical *Trichosporon* isolates (15). Similarly, our data revealed that ravuconazole demonstrates good activity against *Trichosporon* isolates, with MIC values only higher than those of voriconazole and isavuconazole. The current European Confederation of Medical Mycology guidelines recommend voriconazole and posaconazole as first-line treatments for invasive *Trichosporon* infections. However, the clinical use of voriconazole is often limited by adverse effects, including visual hallucinations and hepatotoxicity, while both drugs are associated with significant drug interactions (5, 29). In the current study, both isavuconazole and ravuconazole showed favorable *in vitro* activity against *Trichosporon* isolates, warranting further research to explore their *in vivo* efficacy.

Many studies have investigated the concordance between various AST methods; however, the results have been inconclusive, with frequent discrepancies in MIC values. While our data demonstrated that most discrepancies were attributable to lower amphotericin B and itraconazole MICs and higher voriconazole MICs observed with the SYO assay compared to the CLSI BMD method, the overall performance of the SYO assay showed good agreement within ±2 twofold dilutions. The CA for all antifungal agents exceeded 92% when evaluated using either the L-ECOFFs or CLSI-established ECVs (Table 3). However, the factors contributing to these discrepancies remain unclear and require further investigation using larger datasets.

Our findings indicate that the L-ECOFFs for *T. asahii* were aligned with CLSI ECVs, with differences limited to one- to twofold dilutions, except for amphotericin B. Minor discrepancies in ECVs across studies are common, owing to inherent methodological differences in BMD testing. The observed variation in voriconazole L-ECOFF between our study and that of Francisco et al. (24) may stem from differences in isolate sources, collection periods, and regional factors. Our isolates were primarily obtained from bloodstream infections of patients in Taiwan (2019–2020), whereas those of Francisco et al. were predominantly obtained from urine samples in Brazil (1997–2018). Additionally, emerging trends of increasing azole MICs over time have been reported (25), supporting the need for ongoing surveillance. Further studies incorporating larger numbers of isolates are warranted to evaluate the correlation between phenotypic variability and triazole resistance mechanisms.

Our study has certain limitations. First, the number and diversity of the non-*T. asahii* isolates were relatively limited, potentially influencing the representation of our findings. Second, the ECV for voriconazole, the preferred treatment for *Trichosporon* disease, has not yet been established by the CLSI, preventing further agreement analysis. Third, our study included only a few fluconazole non-WT isolates, which limited our ability to assess the performance of both methods. Further investigations incorporating a larger number of non-WT isolates are necessary to comprehensively evaluate the reliability of each method for detecting antifungal resistance.

In conclusion, isavuconazole and ravuconazole demonstrated favorable *in vitro* activity against clinical *Trichosporon* isolates, while the SYO assay exhibited good overall concordance with the CLSI BMD method. Further studies are needed to confirm these findings and to explore their clinical implications.

## SUPPLEMENTAL MATERIAL

Tables S1 to S3; Figure S1 and S2 jcm.00319-25-s0001.docx *In vitro* activity of antifungals against 43 non-*asahii Trichosporon* isolates. Distribution of MICs of *T. asahii* isolates was analyzed using the ECOFFinder software.

ASM does not own the copyrights to Supplemental Material that may be linked to, or accessed through, an article. The authors have granted ASM a non-exclusive, world-wide license to publish the Supplemental Material files. Please contact the corresponding author directly for reuse.

## References

[1] Arastehfar A, de Almeida Júnior JN, Perlin DS, Ilkit M, Boekhout T, Colombo AL. 2021. Multidrug-resistant Trichosporon species: underestimated fungal pathogens posing imminent threats in clinical settings. Crit Rev Microbiol 47:679–698. doi:10.1080/1040841X.2021.1921695 34115962

[2] Sugita T, Nishikawa A, Ichikawa T, Ikeda R, Shinoda T. 2000. Isolation of Trichosporon asahii from environmental materials. Med Mycol 38:27–30. doi:10.1080/mmy.38.1.27.30 10746224

[3] Colombo AL, Padovan ACB, Chaves GM. 2011. Current knowledge of Trichosporon spp. and trichosporonosis. Clin Microbiol Rev 24:682–700. doi:10.1128/CMR.00003-11 21976604 PMC3194827

[4] Haupt HM, Merz WG, Beschorner WE, Vaughan WP, Saral R. 1983. Colonization and infection with Trichosporon species in the immunosuppressed host. J Infect Dis 147:199–203. doi:10.1093/infdis/147.2.199 6827136

[5] Chen SC-A, Perfect J, Colombo AL, Cornely OA, Groll AH, Seidel D, Albus K, de Almedia JN Jr, Garcia-Effron G, Gilroy N, et al.. 2021. Global guideline for the diagnosis and management of rare yeast infections: an initiative of the ECMM in cooperation with ISHAM and ASM. Lancet Infect Dis 21:e375–e386. doi:10.1016/S1473-3099(21)00203-6 34419208

[6] Kuo S-H, Lu P-L, Chen Y-C, Ho M-W, Lee C-H, Chou C-H, Lin S-Y. 2021. The epidemiology, genotypes, antifungal susceptibility of Trichosporon species, and the impact of voriconazole on Trichosporon fungemia patients. J Formos Med Assoc 120:1686–1694. doi:10.1016/j.jfma.2020.12.007 33358563

[7] Lin S-Y, Lu P-L, Tan BH, Chakrabarti A, Wu U-I, Yang J-H, Patel AK, Li RY, Watcharananan SP, Liu Z, Chindamporn A, Tan AL, Sun P-L, Hsu L-Y, Chen Y-C, Asia Fungal Working Group (AFWG). 2019. The epidemiology of non-Candida yeast isolated from blood: the Asia surveillance study. Mycoses 62:112–120. doi:10.1111/myc.12852 30230062 PMC7379604

[8] Nobrega de Almeida J Jr, Moreno L, Francisco EC, Noronha Marques G, Mendes AV, Barberino MG, Colombo AL. 2021. Trichosporon asahii superinfections in critically ill COVID-19 patients overexposed to antimicrobials and corticosteroids. Mycoses 64:817–822. doi:10.1111/myc.13333 34091966 PMC8242571

[9] de Almeida Júnior JN, Hennequin C. 2016. Invasive Trichosporon infection: a systematic review on a re-emerging fungal pathogen. Front Microbiol 7:1629. doi:10.3389/fmicb.2016.01629 27799926 PMC5065970

[10] Ellsworth M, Ostrosky-Zeichner L. 2020. Isavuconazole: mechanism of action, clinical efficacy, and resistance. J Fungi (Basel) 6:324. doi:10.3390/jof6040324 33260353 PMC7712939

[11] Cornely OA, Mullane KM, Ostrosky-Zeichner L, Maher RM, Croos-Dabrera R, Lu Q, Lademacher C, Perfect JR, Oren I, Schmitt-Hoffmann A-H, Giladi M, Marty FM, Rahav G. 2018. Isavuconazole for treatment of rare invasive fungal diseases. Mycoses 61:518–533. doi:10.1111/myc.12778 29611246

[12] Feugray G, Krzisch D, Dehais M, Razakandrainibe R, Gargala G, Favennec L, Lepretre S, Camus V, Costa D. 2019. Successful treatment of Trichosporon asahii fungemia with isavuconazole in a patient with hematologic malignancies. Infect Drug Resist 12:2015–2018. doi:10.2147/IDR.S211148 31372009 PMC6628197

[13] Almulhim AM, Vellozzi-Averhoff CM, Howard-Anderson J, Babiker A, Kraft CS. 2020. Photo quiz: bilateral necrotizing pneumonia in a 30-year-old woman, a hairy situation. J Clin Microbiol 58:e00451-20. doi:10.1128/JCM.00451-20 33087544 PMC7587113

[14] Hazirolan G, Canton E, Sahin S, Arikan-Akdagli S. 2013. Head-to-head comparison of inhibitory and fungicidal activities of fluconazole, itraconazole, voriconazole, posaconazole, and isavuconazole against clinical isolates of Trichosporon asahii. Antimicrob Agents Chemother 57:4841–4847. doi:10.1128/AAC.00850-13 23877683 PMC3811412

[15] Francisco EC, Dieleman C, Hagen F, Colombo AL, Trichosporon Brazilian Network. 2023. In vitro activity of isavuconazole against clinically relevant Trichosporon species: a comparative evaluation of EUCAST broth microdilution and MIC test strip methods. J Antimicrob Chemother 78:817–822. doi:10.1093/jac/dkad016 36702754

[16] Dong J, Liang G, Zheng H, Kan S, Song N, Zhang M, Liu W. 2021. In vitro activity of ravuconazole against Candida auris and vaginal Candida isolates. Mycoses 64:651–655. doi:10.1111/myc.13260 33609301

[17] Clinical and Laboratory Standards Institute. 2017. Reference method for broth dilution antifungal susceptibility testing of yeasts. 4th ed. CLSI, Wayne, PA, USA.

[18] Subcommittee on Antifungal Susceptibility Testing of the EECfAST. 2008. EUCAST technical note on the method for the determination of broth dilution minimum inhibitory concentrations of antifungal agents for conidia–forming moulds. Clin Microbiol Infect 14:982–984. doi:10.1111/j.1469-0691.2008.02086.x 18828858

[19] Israel S, Perlman A, Moran-Gilad J, Korem M. 2021. Direct fluconazole disk susceptibility testing for Candida glabrata-positive blood cultures. J Clin Microbiol 59:e0031121. doi:10.1128/JCM.00311-21 33883184 PMC8218755

[20] Kritikos A, Neofytos D, Khanna N, Schreiber PW, Boggian K, Bille J, Schrenzel J, Mühlethaler K, Zbinden R, Bruderer T, Goldenberger D, Pfyffer G, Conen A, Van Delden C, Zimmerli S, Sanglard D, Bachmann D, Marchetti O, Lamoth F, Fungal Infection Network of Switzerland (FUNGINOS). 2018. Accuracy of Sensititre YeastOne echinocandins epidemiological cut-off values for identification of FKS mutant Candida albicans and Candida glabrata: a ten year national survey of the Fungal Infection Network of Switzerland (FUNGINOS). Clin Microbiol Infect 24:1214. doi:10.1016/j.cmi.2018.05.012 29909005

[21] Eddouzi J, Lohberger A, Vogne C, Manai M, Sanglard D. 2013. Identification and antifungal susceptibility of a large collection of yeast strains isolated in Tunisian hospitals. Med Mycol 51:737–746. doi:10.3109/13693786.2013.800239 23768242

[22] Cai W, Ruan Q, Li J, Lin L, Xi L, Sun J, Lu S. 2023. Fungal spectrum and susceptibility against nine antifungal agents in 525 deep fungal infected cases. Infect Drug Resist 16:4687–4696. doi:10.2147/IDR.S403863 37484904 PMC10362860

[23] Taverna CG, Córdoba S, Murisengo OA, Vivot W, Davel G, Bosco-Borgeat ME. 2014. Molecular identification, genotyping, and antifungal susceptibility testing of clinically relevant Trichosporon species from Argentina. Med Mycol 52:356–366. doi:10.1093/mmy/myt029 24682113

[24] Francisco EC, de Almeida Junior JN, de Queiroz Telles F, Aquino VR, Mendes AVA, de Andrade Barberino MGM, de Tarso O Castro P, Guimarães T, Hahn RC, Padovan ACB, Chaves GM, Colombo AL. 2019. Species distribution and antifungal susceptibility of 358 Trichosporon clinical isolates collected in 24 medical centres. Clin Microbiol Infect 25:909. doi:10.1016/j.cmi.2019.03.026 30991116

[25] Espinel-Ingroff A, Pfaller M, Messer SA, Knapp CC, Killian S, Norris HA, Ghannoum MA. 1999. Multicenter comparison of the Sensititre YeastOne colorimetric antifungal panel with the national committee for clinical laboratory standards M27-A reference method for testing clinical isolates of common and emerging Candida spp., Cryptococcus spp., and other yeasts and yeast-like organisms. J Clin Microbiol 37:591–595. doi:10.1128/JCM.37.3.591-595.1999 9986817 PMC84481

[26] Pfaller MA, Espinel-Ingroff A, Jones RN. 2004. Clinical evaluation of the Sensititre YeastOne colorimetric antifungal plate for antifungal susceptibility testing of the new triazoles voriconazole, posaconazole, and ravuconazole. J Clin Microbiol 42:4577–4580. doi:10.1128/JCM.42.10.4577-4580.2004 15472311 PMC522344

[27] CLSI. 2022. CLSI supplement M57S. Epidemiological cutoff values for antifungal susceptibility testing. 4th ed. Clinical and Laboratory Standards Institute.

[28] Francisco EC, de Almeida Junior JN, Queiroz-Telles F, Aquino VR, Mendes AVA, de Oliveira Silva M, Castro P de TOE, Guimarães T, Ponzio V, Hahn RC, Chaves GM, Colombo AL. 2021. Correlation of Trichosporon asahii genotypes with anatomical sites and antifungal susceptibility profiles: data analyses from 284 isolates collected in the last 22 years across 24 medical centers. Antimicrob Agents Chemother 65:e01104-20. doi:10.1128/AAC.01104-20 33318016 PMC8092518

[29] Azanza JR, Mensa J, Barberán J, Vázquez L, Pérez de Oteyza J, Kwon M, Yáñez L, Aguado JM, Cubillo Gracian A, Solano C, Ruiz Camps I, Fortún J, Salavert Lletí M, Gudiol C, Olave Rubio T, García-Vidal C, Rovira Tarrats M, Suárez-Lledó Grande M, González-Sierra P, Dueñas Gutiérrez C. 2023. Recommendations on the use of azole antifungals in hematology-oncology patients. Rev Esp Quimioter 36:236–258. doi:10.37201/req/013.2023 37017117 PMC10238801

