# Supplementary material for: In vitro activity of isavuconazole, ravuconazole, and comparison of the Sensititre YeastOne and CLSI broth microdilution methods against clinical isolates of Trichosporon species
Source: J Clin Microbiol. 2025 May 12;63(6):e00319-25. doi: 10.1128/jcm.00319-25 (PMC12153333; doi:10.1128/jcm.00319-25)
Supplement: Tables S1 to S3; Figure S1 and S2 — In vitro activity of antifungals against 43 non-asahii Trichosporon isolates. Distribution of MICs of T. asahii isolates was analyzed using the ECOFFinder software. [file jcm.00319-25-s0001.docx]

**Table S1. Distribution of samples from different sites**

| **Sample types** | **Details** | **Numbers (%)** |
| --- | --- | --- |
| Blood |  | 105 (39.3%) |
| Respiratory tract sample | Nasal/throat swab, sputum, bronchial washing, bronchoscope specimen, endotracheal aspiration, pleural effusion | 52 (19.5%) |
| Urine |  | 37 (13.9%) |
| Superficial wound | Wound pus, bedsore, eye, nail | 29 (10.9%) |
| Ascites | Ascites, peritoneal fluid | 15 (5.6%) |
| Feces |  | 3 (1.1%) |
| Others | Abscess, bile, tissue, body fluid, CSF, CVC, double lumen tip, hemovac drain tube, PTCD drainage, soft tissue, synovial fluid | 26 (9.7%) |

CSF, cerebrospinal fluid; CVC, central venous catheter; PTCD, percutaneous transhepatic biliary drainage

**Table S2. *In vitro* activity of amphotericin B, fluconazole, itraconazole, voriconazole, posaconazole, ravuconazole, and isavuconazole against 43 *Non-asahii* *Trichosporon* isolates using the reference CLSI broth microdilution method**

|  |  | Number of isolates per MIC value (mg/L) | | | | | | | | | | | | MIC range | MIC_50_ | MIC_90_ | Mode | GM |
| --- | --- | --- | --- | --- | --- | --- | --- | --- | --- | --- | --- | --- | --- | --- | --- | --- | --- | --- |
| **Amphotericin B** | Number | ≦0.015 0.03 0.06 0.12 0.25 0.5 1 2 4 8 16 32 | | | | | | | | | | | | mg/L | | | | |
| *Non-asahii Trichosporon* | 43 |  |  |  | 1 | 1 | 12 | 27 | 2 |  |  |  |  | 0.12-2.0 | 1 | 1 | 1 | 0.78 |
| *T. montevideense* | 11 |  |  |  |  | 1 | 3 | 6 | 1 |  |  |  |  | 0.25-2.0 | 1 | 1 | 1 | 0.78 |
| *T. mucoides* | 11 |  |  |  |  |  | 3 | 8 |  |  |  |  |  | 0.5-1.0 | 1 | 1 | 1 | 0.83 |
| *T. dermatis* | 6 |  |  |  |  |  | 1 | 5 |  |  |  |  |  | 0.5-1.0 | 1 | 1 | 1 | 0.89 |
| *T. dohaense* | 1 |  |  |  |  |  | 1 |  |  |  |  |  |  | 0.5 | ND | ND | ND | 0.5 |
| *T. faecale* | 7 |  |  |  |  |  | 2 | 5 |  |  |  |  |  | 0.5-1.0 | 1 | 1 | 1 | 0.82 |
| *T. inkin* | 1 |  |  |  |  |  | 1 |  |  |  |  |  |  | 0.5 | ND | ND | ND | 0.5 |
| *T. japonicum* | 3 |  |  |  |  |  | 1 | 2 |  |  |  |  |  | 0.5-1.0 | ND | ND | ND | 0.79 |
| *T. jirovecii* | 1 |  |  |  | 1 |  |  |  |  |  |  |  |  | 0.12 | ND | ND | ND | 0.12 |
| *T. mycotoxinivorans* | 2 |  |  |  |  |  |  | 1 | 1 |  |  |  |  | 1.0-2.0 | ND | ND | ND | 1.41 |
| **Fluconazole** |  |  |  |  |  |  |  |  |  |  |  |  |  |  |  |  |  |  |
| *Non-asahii Trichosporon* | 43 |  |  |  |  |  |  | 10 | 13 | 16 | 3 | 1 |  | 1.0-16.0 | 2 | 4 | 4 | 2.55 |
| *T. montevideense* | 11 |  |  |  |  |  |  | 3 | 8 |  |  |  |  | 1.0-2.0 | 2 | 2 | 2 | 1.66 |
| *T. mucoides* | 11 |  |  |  |  |  |  |  | 2 | 6 | 3 |  |  | 2.0-8.0 | 4 | 8 | 4 | 4.26 |
| *T. dermatis* | 6 |  |  |  |  |  |  | 2 | 1 | 3 |  |  |  | 1.0-4.0 | 4 | 4 | 4 | 2.24 |
| *T. dohaense* | 1 |  |  |  |  |  |  | 1 |  |  |  |  |  | 1 | ND | ND | ND | 1 |
| *T. faecale* | 7 |  |  |  |  |  |  | 1 |  | 6 |  |  |  | 1.0-4.0 | 4 | 4 | 4 | 3.28 |
| *T. inkin* | 1 |  |  |  |  |  |  |  | 1 |  |  |  |  | 2 | ND | ND | ND | 2 |
| *T. japonicum* | 3 |  |  |  |  |  |  | 2 | 1 |  |  |  |  | 1.0-2.0 | ND | ND | ND | 1.26 |
| *T. jirovecii* | 1 |  |  |  |  |  |  | 1 |  |  |  |  |  | 1 | ND | ND | ND | 1 |
| *T. mycotoxinivorans* | 2 |  |  |  |  |  |  |  |  | 1 |  | 1 |  | 4.0-16.0 | ND | ND | ND | 8 |
| **Itraconazole** |  |  |  |  |  |  |  |  |  |  |  |  |  |  |  |  |  |  |
| *Non-asahii Trichosporon* | 43 |  |  | 2 | 3 | 10 | 26 | 2 |  |  |  |  |  | 0.06-1.0 | 0.5 | 0.5 | 0.5 | 0.36 |
| *T. montevideense* | 11 |  |  |  |  | 5 | 6 |  |  |  |  |  |  | 0.25-0.5 | 0.5 | 0.5 | 0.5 | 0.36 |
| *T. mucoides* | 11 |  |  | 1 |  | 1 | 8 | 1 |  |  |  |  |  | 0.06-1.0 | 0.5 | 0.5 | 0.5 | 0.41 |
| *T. dermatis* | 6 |  |  | 1 | 1 | 1 | 3 |  |  |  |  |  |  | 0.06-0.5 | 0.5 | 0.5 | 0.5 | 0.25 |
| *T. dohaense* | 1 |  |  |  |  |  | 1 |  |  |  |  |  |  | 0.5 | ND | ND | ND | 0.5 |
| *T. faecale* | 7 |  |  |  | 1 |  | 6 |  |  |  |  |  |  | 0.12-0.5 | 0.5 | 0.5 | 0.5 | 0.41 |
| *T. inkin* | 1 |  |  |  |  |  | 1 |  |  |  |  |  |  | 0.5 | ND | ND | ND | 0.5 |
| *T. japonicum* | 3 |  |  |  | 1 | 2 |  |  |  |  |  |  |  | 0.12-0.25 | ND | ND | ND | 0.2 |
| *T. jirovecii* | 1 |  |  |  |  | 1 |  |  |  |  |  |  |  | 0.25 | ND | ND | ND | 0.25 |
| *T. mycotoxinivorans* | 2 |  |  |  |  |  | 1 | 1 |  |  |  |  |  | 0.5-0.1 | ND | ND | ND | 0.71 |
| **Posaconazole** |  |  |  |  |  |  |  |  |  |  |  |  |  |  |  |  |  |  |
| *Non-asahii Trichosporon* | 43 |  | 1 | 4 | 5 | 23 | 10 |  |  |  |  |  |  | 0.03-0.5 | 0.25 | 0.5 | 0.25 | 0.22 |
| *T. montevideense* | 11 |  | 1 | 1 | 2 | 7 |  |  |  |  |  |  |  | 0.03-0.25 | 0.25 | 0.25 | 0.25 | 0.16 |
| *T. mucoides* | 11 |  |  | 1 | 1 | 8 | 1 |  |  |  |  |  |  | 0.06-0.5 | 0.25 | 0.25 | 0.25 | 0.22 |
| *T. dermatis* | 6 |  |  | 1 |  | 3 | 2 |  |  |  |  |  |  | 0.06-0.5 | 0.25 | 0.5 | 0.25 | 0.25 |
| *T. dohaense* | 1 |  |  |  |  | 1 |  |  |  |  |  |  |  | 0.25 | ND | ND | ND | 0.25 |
| *T. faecale* | 7 |  |  |  |  | 4 | 3 |  |  |  |  |  |  | 0.25-0.5 | 0.25 | 0.5 | 0.25 | 0.34 |
| *T. inkin* | 1 |  |  |  |  |  | 1 |  |  |  |  |  |  | 0.5 | ND | ND | ND | 0.5 |
| *T. japonicum* | 3 |  |  | 1 | 1 |  | 1 |  |  |  |  |  |  | 0.06-0.5 | ND | ND | ND | 0.15 |
| *T. jirovecii* | 1 |  |  |  | 1 |  |  |  |  |  |  |  |  | 0.12 | ND | ND | ND | 0.12 |
| *T. mycotoxinivorans* | 2 |  |  |  |  |  | 2 |  |  |  |  |  |  | 0.5 | ND | ND | ND | 0.5 |
| **Voriconazole** |  |  |  |  |  |  |  |  |  |  |  |  |  |  |  |  |  |  |
| *Non-asahii Trichosporon* | 43 | 6 | 18 | 10 | 4 | 3 | 2 |  |  |  |  |  |  | 0.015-0.5 | 0.03 | 0.25 | 0.03 | 0.05 |
| *T. montevideense* | 11 | 2 | 8 | 1 |  |  |  |  |  |  |  |  |  | 0.015-0.06 | 0.03 | 0.03 | 0.03 | 0.03 |
| *T. mucoides* | 11 |  | 4 | 2 | 2 | 3 |  |  |  |  |  |  |  | 0.03-0.25 | 0.06 | 0.25 | 0.03 | 0.08 |
| *T. dermatis* | 6 |  | 1 | 3 | 2 |  |  |  |  |  |  |  |  | 0.03-0.12 | 0.06 | 0.12 | 0.06 | 0.07 |
| *T. dohaense* | 1 | 1 |  |  |  |  |  |  |  |  |  |  |  | 0.015 | ND | ND | ND | 0.015 |
| *T. faecale* | 7 |  | 3 | 4 |  |  |  |  |  |  |  |  |  | 0.03-0.06 | 0.06 | 0.06 | 0.06 | 0.04 |
| *T. inkin* | 1 | 1 |  |  |  |  |  |  |  |  |  |  |  | 0.015 | ND | ND | ND | 0.015 |
| *T. japonicum* | 3 | 2 | 1 |  |  |  |  |  |  |  |  |  |  | 0.015-0.03 | ND | ND | ND | 0.02 |
| *T. jirovecii* | 1 |  | 1 |  |  |  |  |  |  |  |  |  |  | 0.03 | ND | ND | ND | 0.03 |
| *T. mycotoxinivorans* | 2 |  |  |  |  |  | 2 |  |  |  |  |  |  | 0.5 | ND | ND | ND | 0.5 |
| **Ravconazole** |  |  |  |  |  |  |  |  |  |  |  |  |  |  |  |  |  |  |
| *Non-asahii Trichosporon* | 43 | 2 | 13 | 3 | 5 | 10 | 9 | 1 |  |  |  |  |  | 0.008-1 | 0.12 | 0.5 | 0.03 | 0.11 |
| *T. montevideense* | 11 | 1 | 7 |  | 2 | 1 |  |  |  |  |  |  |  | 0.015-0.5 | 0.03 | 0.12 | 0.03 | 0.05 |
| *T. mucoides* | 11 |  | 1 |  | 1 | 5 | 4 |  |  |  |  |  |  | 0.03-0.5 | 0.25 | 0.5 | 0.25 | 0.25 |
| *T. dermatis* | 6 |  | 1 | 1 | 1 | 2 | 1 |  |  |  |  |  |  | 0.03-0.5 | 0.25 | 0.5 | 0.25 | 0.14 |
| *T. dohaense* | 1 | 1 |  |  |  |  |  |  |  |  |  |  |  | 0.008 | ND | ND | ND | 0.008 |
| *T. faecale* | 7 |  |  | 1 | 1 | 3 | 2 |  |  |  |  |  |  | 0.06-0.5 | 0.25 | 0.5 | 0.25 | 0.22 |
| *T. inkin* | 1 |  | 1 |  |  |  |  |  |  |  |  |  |  | 0.03 | ND | ND | ND | 0.03 |
| *T. japonicum* | 3 |  | 3 |  |  |  |  |  |  |  |  |  |  | 0.03 | ND | ND | ND | 0.03 |
| *T. jirovecii* | 1 |  |  | 1 |  |  |  |  |  |  |  |  |  | 0.06 | ND | ND | ND | 0.06 |
| *T. mycotoxinivorans* | 2 |  |  |  |  |  | 1 | 1 |  |  |  |  |  | 0.5-1 | ND | ND | ND | 0.71 |
| **Isavuconazole** |  |  |  |  |  |  |  |  |  |  |  |  |  |  |  |  |  |  |
| *Non-asahii Trichosporon* | 43 | 3 | 11 | 8 | 10 | 8 | 2 |  | 1 |  |  |  |  | 0.015-2 | 0.06 | 0.25 | 0.03 | 0.08 |
| *T. montevideense* | 11 |  | 8 | 2 | 1 |  |  |  |  |  |  |  |  | 0.03-0.12 | 0.03 | 0.06 | 0.03 | 0.04 |
| *T. mucoides* | 11 |  | 1 | 1 | 5 | 3 | 1 |  |  |  |  |  |  | 0.03-0.5 | 0.12 | 0.25 | 0.12 | 0.14 |
| *T. dermatis* | 6 |  | 1 | 2 | 2 | 1 |  |  |  |  |  |  |  | 0.03-0.25 | 0.12 | 0.25 | 0.06 | 0.09 |
| *T. dohaense* | 1 | 1 |  |  |  |  |  |  |  |  |  |  |  | 0.015 | ND | ND | ND | 0.015 |
| *T. faecale* | 7 |  |  | 1 | 2 | 4 |  |  |  |  |  |  |  | 0.06-0.25 | 0.25 | 0.25 | 0.25 | 0.17 |
| *T. inkin* | 1 |  |  | 1 |  |  |  |  |  |  |  |  |  | 0.06 | ND | ND | ND | 0.06 |
| *T. japonicum* | 3 | 1 | 1 | 1 |  |  |  |  |  |  |  |  |  | 0.015-0.06 | ND | ND | ND | 0.03 |
| *T. jirovecii* | 1 | 1 |  |  |  |  |  |  |  |  |  |  |  | 0.015 | ND | ND | ND | 0.015 |
| *T. mycotoxinivorans* | 2 |  |  |  |  |  | 1 |  | 1 |  |  |  |  | 0.5-2 | ND | ND | ND | 1 |

MIC, minimal inhibitory concentration; GM, geometric mean; ND, non-determined

**Table S3. MIC ranges, MIC_50_, MIC_90_ values, and GM MIC for *Trichosporon* species obtained using the SYO assay and the agreement between the SYO and the CLSI BMD methods**

|  | |  |  | Sensititre YeastOne | | | | |  |  | No. (%) of isolates for which MICs determined by YeastOne differed from  MICs determined by the CLSI BMD method at the indicated dilution | | | | | | | | |
| --- | --- | --- | --- | --- | --- | --- | --- | --- | --- | --- | --- | --- | --- | --- | --- | --- | --- | --- | --- |
|  | | No. | | Range | MIC_50_ | MIC_90_ | Mode MIC | GM |  |  | ≧3 | 2 | 1 | 0 | -1 | -2 | ≦-3 | Within ±1 | Within ±2 |
| **Amphotericin B** | |  |  |  |  |  |  |  |  |  |  |  |  |  |  |  |  |  |  |
|  | *Non-asahii Trichosporon* | 43 |  | 0.12-2.0 | 0.5 | 1 | 0.5 | 0.47 |  |  | 1 (2.3) | 0 | 2 (4.7) | 13 (30.2) | 19 (44.2) | 6 (14.0) | 2 (4.7) | 34 (79.1) | 40 (93.0) |
|  | *T. montevideense* | 11 |  | 0.25-1.0 | 0.25 | 1 | 0.25 | 0.44 |  |  | 0 | 0 | 0 | 4 (36.4) | 5 (45.5) | 2 (18.2) | 0 | 9 (81.8) | 11 (100) |
|  | *T. mucoides* | 11 |  | 0.25-1.0 | 0.5 | 1 | 0.5 | 0.5 |  |  | 0 | 0 | 0 | 4 (36.4) | 6 (54.5) | 1 (9.1) | 0 | 10 (90.9) | 11 (100) |
|  | *T. dermatis* | 6 |  | 0.12-0.5 | 0.25 | 0.5 | 0.12 | 0.22 |  |  | 0 | 0 | 0 | 0 | 2 (33.3) | 2 (33.3) | 2 (33.3) | 2 (33.3) | 4 (66.7) |
|  | *T. dohaense* | 1 |  | 0.5 | ND | ND | ND | 0.5 |  |  | 0 | 0 | 0 | 1 (100) | 0 | 0 | 0 | 1 (100) | 1 (100) |
|  | *T. faecale* | 7 |  | 0.25-2.0 | 0.5 | 2 | 0.25 | 0.55 |  |  | 0 | 0 | 2 (28.6) | 0 | 4 (57.1) | 1 (14.3) | 0 | 6 (85.7) | 7 (100) |
|  | *T. inkin* | 1 |  | 0.25 | ND | ND | ND | 0.25 |  |  | 0 | 0 | 0 | 0 | 1 (100) | 0 | 0 | 1 (100) | 1 (100) |
|  | *T. japonicum* | 3 |  | 0.5-1.0 | ND | ND | ND | 0.71 |  |  | 0 | 0 | 0 | 3 (100) | 0 | 0 | 0 | 3 (100) | 3 (100) |
|  | *T. jirovecii* | 1 |  | 1 | ND | ND | ND | 1 |  |  | 1 (100) | 0 | 0 | 0 | 0 | 0 | 0 | 0 | 0 |
|  | *T. mycotoxinivorans* | 2 |  | 1 | ND | ND | ND | 1 |  |  | 0 | 0 | 0 | 1 (50) | 1 (50) | 0 | 0 | 2 (100) | 2 (100) |
| **Fluconazole** | |  |  |  |  |  |  |  |  |  |  |  |  |  |  |  |  |  |  |
|  | *Non-asahii Trichosporon* | 43 |  | 0.12-64.0 | 4 | 8 | 4 | 2.8 |  |  | 0 | 4 (9.3) | 11 (25.6) | 20 (46.5) | 5 (11.6) | 2 (4.7) | 1 (2.3) | 36 (83.7) | 42 (97.7) |
|  | *T. montevideense* | 11 |  | 0.12-4.0 | 2 | 2 | 2 | 1.28 |  |  | 0 | 0 | 2 (18.2) | 6 (54.5) | 2 (18.2) | 0 | 1 (9.1) | 10 (90.9) | 10 (90.9) |
|  | *T. mucoides* | 11 |  | 2.0-4.0 | 2 | 4 | 2 | 2.74 |  |  | 0 | 0 | 0 | 6 (54.5) | 3 (27.3) | 2 (18.2) | 0 | 9 (81.8) | 11 (100) |
|  | *T. dermatis* | 6 |  | 2.0-4.0 | 4 | 4 | 4 | 3.56 |  |  | 0 | 1 (16.7) | 2 (33.3) | 3 (50) | 0 | 0 | 0 | 5 (83.3) | 6 (100) |
|  | *T. dohaense* | 1 |  | 4 | ND | ND | ND | 4 |  |  | 0 | 1 (100) | 0 | 0 | 0 | 0 | 0 | 0 | 1 (100) |
|  | *T. faecale* | 7 |  | 4.0-8.0 | 4 | 8 | 4 | 5.38 |  |  | 0 | 1 (14.3) | 3 (42.9) | 3 (42.9) | 0 | 0 | 0 | 6 (85.7) | 7 (100) |
|  | *T. inkin* | 1 |  | 4 | ND | ND | ND | 4 |  |  | 0 | 0 | 1 (100) | 0 | 0 | 0 | 0 | 1 (100) | 1 (100) |
|  | *T. japonicum* | 3 |  | 1.0-4.0 | ND | ND | ND | 2.00 |  |  | 0 | 0 | 2 (66.7) | 1 (33.3) | 0 | 0 | 0 | 3 (100) | 3 (100) |
|  | *T. jirovecii* | 1 |  | 1 | ND | ND | ND | 1 |  |  | 0 | 0 | 0 | 1 (100) | 0 | 0 | 0 | 1 (100) | 1 (100) |
|  | *T. mycotoxinivorans* | 2 |  | 8.0-64.0 | ND | ND | ND | 22.63 |  |  | 0 | 1 (50) | 1 (50) | 0 | 0 | 0 | 0 | 1 (50) | 2 (100) |
| **Itraconazole** | |  |  |  |  |  |  |  |  |  |  |  |  |  |  |  |  |  |  |
|  | *Non-asahii Trichosporon* | 43 |  | 0.008-0.5 | 0.12 | 0.25 | 0.12 | 0.11 |  |  | 0 | 1 (2.3) | 2 (4.7) | 8 (18.6) | 8 (18.6) | 15 (34.9) | 9 (20.9) | 18 (41.9) | 34 (79.1) |
|  | *T. montevideense* | 11 |  | 0.008-0.25 | 0.12 | 0.12 | 0.12 | 0.05 |  |  | 0 | 0 | 0 | 1 (9.1) | 2 (18.2) | 3 (27.3) | 5 (45.5) | 3 (27.3) | 6 (54.5) |
|  | *T. mucoides* | 11 |  | 0.03-0.25 | 0.12 | 0.25 | 0.12 | 0.1 |  |  | 0 | 0 | 0 | 2 (18.2) | 1 (9.1) | 4 (36.4) | 4 (36.4) | 3 (27.3) | 7 (63.6) |
|  | *T. dermatis* | 6 |  | 0.06-0.12 | 0.12 | 0.12 | 0.12 | 0.10 |  |  | 0 | 0 | 1 (16.7) | 0 | 1 (16.7) | 4 (66.7) | 0 | 2 (33.3) | 6 (100) |
|  | *T. dohaense* | 1 |  | 0.12 | ND | ND | ND | 0.12 |  |  | 0 | 0 | 0 | 0 | 0 | 1 (100) | 0 | 0 | 1 (100) |
|  | *T. faecale* | 7 |  | 0.12-0.5 | 0.25 | 0.5 | 0.25 | 0.3 |  |  | 0 | 1 (14.3) | 0 | 2 (28.6) | 3 (42.9) | 1 (14.3) | 0 | 5 (71.4) | 7 (100) |
|  | *T. inkin* | 1 |  | 0.12 | ND | ND | ND | 0.12 |  |  | 0 | 0 | 0 | 0 | 0 | 1 (100) | 0 | 0 | 1 (100) |
|  | *T. japonicum* | 3 |  | 0.25 | ND | ND | ND | 0.25 |  |  | 0 | 0 | 1 (33.3) | 2 (66.7) | 0 | 0 | 0 | 3 (100) | 3 (100) |
|  | *T. jirovecii* | 1 |  | 0.12 | ND | ND | ND | 0.12 |  |  | 0 | 0 | 0 | 0 | 1 (100) | 0 | 0 | 1 (100) | 1 (100) |
|  | *T. mycotoxinivorans* | 2 |  | 0.25-0.5 | ND | ND | ND | 0.35 |  |  | 0 | 0 | 0 | 1 (50) | 0 | 1 (50) | 0 | 1 (50) | 2 (100) |
| **Posaconazole** | |  |  |  |  |  |  |  |  |  |  |  |  |  |  |  |  |  |  |
|  | *Non-asahii Trichosporon* | 43 |  | 0.008-1.0 | 0.12 | 0.5 | 0.12 | 0.15 |  |  | 0 | 1 (2.3) | 9 (20.9) | 10 (23.3) | 15 (34.9) | 5 (11.6) | 3 (7.0) | 34 (79.1) | 40 (93.0) |
|  | *T. montevideense* | 11 |  | 0.008-0.25 | 0.12 | 0.25 | 0.12 | 0.08 |  |  | 0 | 0 | 2 (18.2) | 2 (18.2) | 5 (45.5) | 0 | 2 (18.2) | 9 (81.8) | 9 (81.8) |
|  | *T. mucoides* | 11 |  | 0.03-0.5 | 0.12 | 0.25 | 0.12 | 0.11 |  |  | 0 | 0 | 1 (9.1) | 2 (18.2) | 5 (45.5) | 2 (18.2) | 1 (9.1) | 8 (72.7) | 10 (90.9) |
|  | *T. dermatis* | 6 |  | 0.12 | 0.12 | 0.12 | 0.12 | 0.12 |  |  | 0 | 0 | 1 (16.7) | 0 | 3 (50) | 2 (33.3) | 0 | 4 (66.7) | 6 (100) |
|  | *T. dohaense* | 1 |  | 0.12 | ND | ND | ND | 0.12 |  |  | 0 | 0 | 0 | 0 | 1 (100) | 0 | 0 | 1 (100) | 1 (100) |
|  | *T. faecale* | 7 |  | 0.25-1.0 | 0.5 | 1 | 0.5 | 0.5 |  |  | 0 | 0 | 4 (57.1) | 3 (42.9) | 0 | 0 | 0 | 7 (100) | 7 (100) |
|  | *T. inkin* | 1 |  | 0.12 | ND | ND | ND | 0.12 |  |  | 0 | 0 | 0 | 0 | 0 | 1 (100) | 0 | 0 | 1 (100) |
|  | *T. japonicum* | 3 |  | 0.25 | ND | ND | ND | 0.25 |  |  | 0 | 1 (33.3) | 1 (33.3) | 0 | 1 (33.3) | 0 | 0 | 2 (66.7) | 3 (100) |
|  | *T. jirovecii* | 1 |  | 0.12 | ND | ND | ND | 0.12 |  |  | 0 | 0 | 0 | 1 (100) | 0 | 0 | 0 | 1 (100) | 1 (100) |
|  | *T. mycotoxinivorans* | 2 |  | 0.5 | ND | ND | ND | 0.5 |  |  | 0 | 0 | 0 | 2 (100) | 0 | 0 | 0 | 2 (100) | 2 (100) |
| **Voriconazole** | |  |  |  |  |  |  |  |  |  |  |  |  |  |  |  |  |  |  |
|  | *Non-asahii Trichosporon* | 43 |  | 0.008-1.0 | 0.06 | 0.25 | 0.12 | 0.07 |  |  | 3 (7.0) | 9 (20.9) | 9 (20.9) | 12 (27.9) | 7 (16.3) | 3 (7.0) | 0 | 28 (65.1) | 40 (93.0) |
|  | *T. montevideense* | 11 |  | 0.008-0.12 | 0.03 | 0.06 | 0.03 | 0.03 |  |  | 0 | 1 (9.1) | 2 (18.2) | 5 (45.5) | 3 (27.3) | 0 | 0 | 10 (90.9) | 11 (100) |
|  | *T. mucoides* | 11 |  | 0.03-0.12 | 0.06 | 0.12 | 0.12 | 0.07 |  |  | 0 | 2 (18.2) | 1 (9.1) | 4 (36.4) | 1 (9.1) | 3 (27.3) | 0 | 6 (54.5) | 11 (100) |
|  | *T. dermatis* | 6 |  | 0.03-0.12 | 0.06 | 0.12 | 0.06 | 0.07 |  |  | 0 | 0 | 2 (33.3) | 2 (33.3) | 2 (33.3) | 0 | 0 | 6 (100) | 6 (100) |
|  | *T. dohaense* | 1 |  | 0.06 | ND | ND | ND | 0.06 |  |  | 0 | 1 (100) | 0 | 0 | 0 | 0 | 0 | 0 | 1 (100) |
|  | *T. faecale* | 7 |  | 0.06-0.25 | 0.12 | 0.25 | 0.12 | 0.15 |  |  | 2 (28.6) | 2 (28.6) | 2 (28.6) | 1 (14.3) | 0 | 0 | 0 | 3 (42.9) | 5 (71.4) |
|  | *T. inkin* | 1 |  | 0.06 | ND | ND | ND | 0.06 |  |  | 0 | 1 (100) | 0 | 0 | 0 | 0 | 0 | 0 | 1 (100) |
|  | *T. japonicum* | 3 |  | 0.06-0.12 | ND | ND | ND | 0.10 |  |  | 1 (33.3) | 2 (66.7) | 0 | 0 | 0 | 0 | 0 | 0 | 2 (66.7) |
|  | *T. jirovecii* | 1 |  | 0.06 | ND | ND | ND | 0.06 |  |  | 0 | 0 | 1 (100) | 0 | 0 | 0 | 0 | 1 (100) | 1 (100) |
|  | *T. mycotoxinivorans* | 2 |  | 0.25-1.0 | ND | ND | ND | 0.5 |  |  | 0 | 0 | 1 (50) | 0 | 1 (50) | 0 | 0 | 2 (100) | 2 (100) |

BMD, broth microdilution; MIC, minimal inhibitory concentration; GM, geometric mean; ND, non-determined; SYO, Sensititre YeastOne


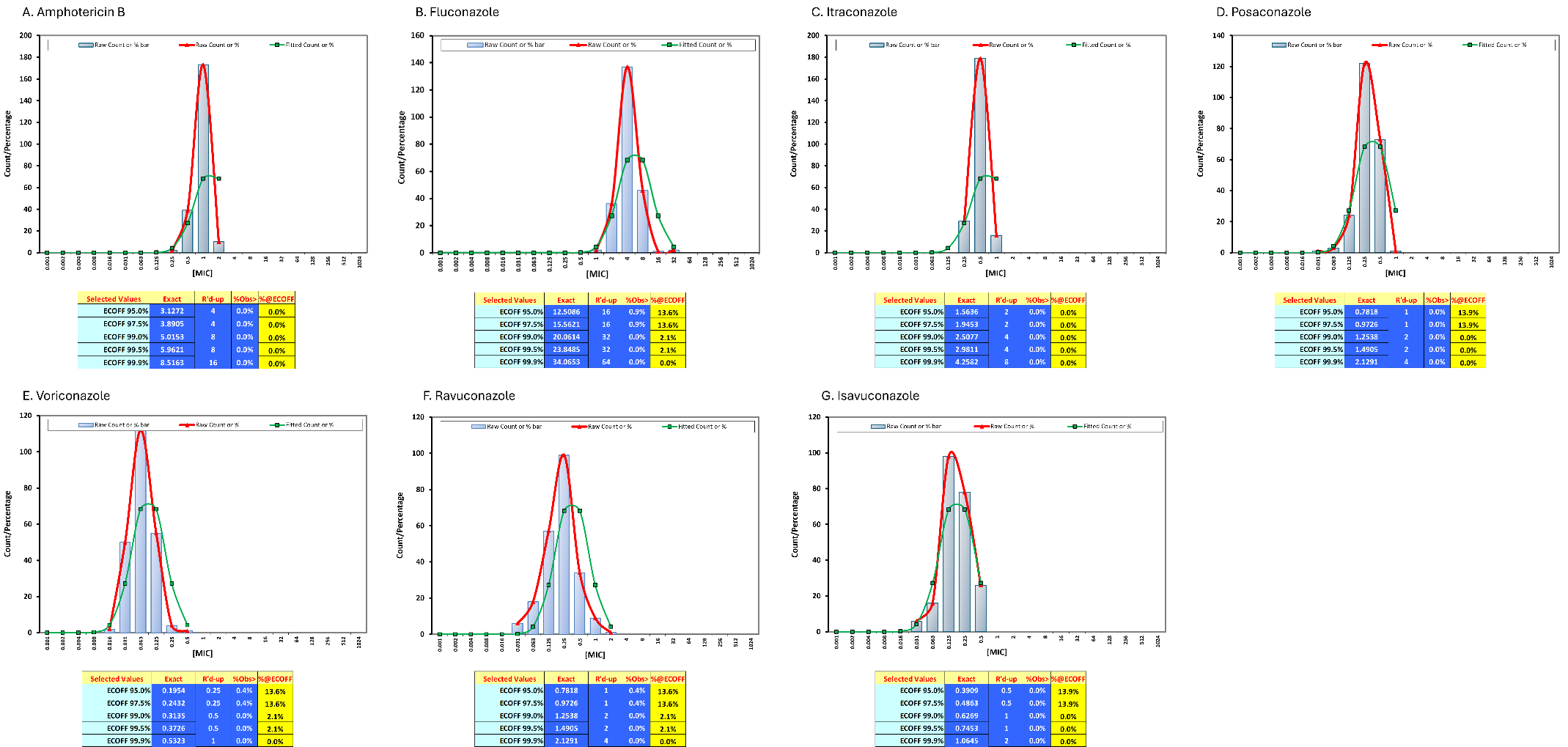


**Figure S1.** Distribution of MICs of 224 *T. asahii* clinical isolates obtained using the CLSI broth microdilution method and analyzed using the ECOFFinder software.


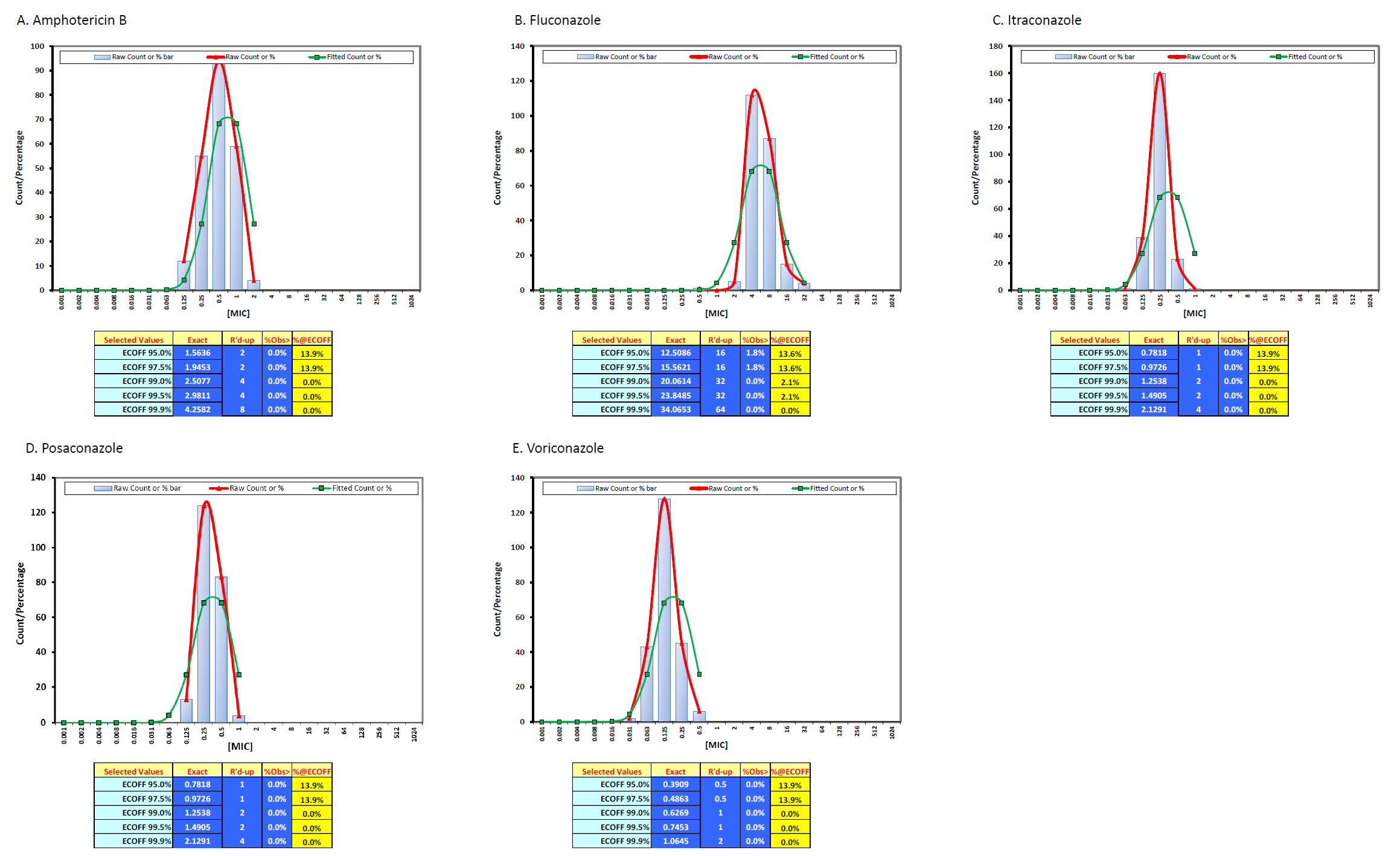


**Figure S2.** Distribution of MICs of 224 *T. asahii* clinical isolates clinical isolates obtained using the Sensititre YeastOne method and analyzed using the ECOFFinder software.
